# Towards appropriate information provision for and decision-making with patients with limited health literacy in hospital-based palliative care in Western countries: a scoping review into available communication strategies and tools for healthcare providers

**DOI:** 10.1186/s12904-019-0421-x

**Published:** 2019-04-12

**Authors:** Janneke Noordman, Liesbeth van Vliet, Menno Kaunang, Maria van den Muijsenbergh, Gudule Boland, Sandra van Dulmen

**Affiliations:** 1 Nivel, Netherlands institute for health services research, P.O. Box 1568, 3500 BN Utrecht, The Netherlands; 2 Department of Primary and Community Care, Radboud university medical center, Radboud Institute for Health Sciences, Nijmegen, The Netherlands; 3 Department of Health, Medical and Neuropsychology, Institute of Psychology, Leiden University, Leiden, The Netherlands; 4 Pharos, Dutch Centre of Expertise on Health Disparities, Utrecht, The Netherlands; 5 Faculty of Health and Social Sciences, University of South-Eastern Norway, Drammen, Norway

**Keywords:** Palliative care, Limited health literacy, Healthcare providers, Hospital, Patients, Review literature

## Abstract

**Background:**

Person-centred palliative care poses high demands on professionals and patients regarding appropriate and effective communication and informed decision-making. This is even more so for patients with limited health literacy, as they lack the necessary skills to find, understand and apply information about their health and healthcare. Recognizing patients with limited health literacy and adapting the communication, information provision and decision-making process to their skills and needs is essential to achieve desired person-centred palliative care. The aim of this study is to summarize available strategies and tools for healthcare providers towards successful communication, information provision and/or shared decision-making in supporting patients with limited health literacy in hospital-based palliative care in Western countries.

**Methods:**

A scoping review was conducted. First, databases PubMed, Embase, CINAHL, and PsycINFO were searched. Next, grey literature was examined using several online databases and by contacting national experts. In addition, all references of included studies were checked.

**Results:**

Five studies were included that showed that there are face-to-face, written as well as online strategies available for healthcare providers to support communication, information provision and, to a lesser extent, (shared) decision-making in palliative care for patients with limited health literacy. Strategies that were mentioned several times were: teach-back method, jargon-free communication and developing and testing materials with patients with limited health literacy, among others. Two supporting tools were found: patient decision aids and question prompt lists.

**Conclusions:**

To guarantee high quality person-centred palliative care, the role of health literacy should be considered. Although there are several strategies available for healthcare providers to facilitate such communication, only few tools are offered. Moreover, the strategies and tools appear not specific for the setting of palliative care, but seem helpful for providers to support the communication, information provision and decision making with patients with limited health literacy in general. Future research should focus on which strategies or tools are (most) effective in supporting patients with limited health literacy in palliative care, and the implementation of these strategies and tools in practice.

**Electronic supplementary material:**

The online version of this article (10.1186/s12904-019-0421-x) contains supplementary material, which is available to authorized users.

## Background

Almost 48% of the European population has limited health literacy (LHL), with a prevalence ranging from 36% in the Netherlands to 62% in Bulgaria [1, 2]. People with LHL lack the necessary skills to find, understand and apply information about health and healthcare [3, 4]. Although LHL is situation-bound and can affect all people, it is more common in the group of low(er) educated persons, males, elderly (65 years or older) and people who judge their health as poor(er) [1]. LHL hampers communication with health professionals and shared decision-making because it affects the ability to ask questions, to understand information, and to reflect and plan ahead [4]. The patients’ skills become even more essential when being confronted with a life-limiting disease that requires preference-sensitive treatment decisions. The great majority (69–82%) of all deaths will be preceded by such a palliative care phase [5]. Hospital-based palliative care interventions are often complex and considered appropriate when they are consistent with patients’ wishes [6, 7]. Achieving such person-centred care relies on effective and high-quality communication between health care professionals and patients about treatment goals and options, as well as on clear information provision about the organization of healthcare and support options for the patient in and outside the hospital [7–9].

Good communication in palliative care poses high demands on health care professionals’ skills and this is especially true when caring for patients with LHL. In general and especially in palliative care, patients experience difficulties in communicating with health care professionals and consequently in making informed care decisions [10–13]. A recent systematic review revealed that health care professionals and patients with COPD want to communicate about palliative care (i.e. advanced care planning), but rarely do so in practice [14]. These difficulties could be the result of e.g. communication skills of the health care professional, insufficient time or LHL skills of the patient.

LHL is a known barrier to patient participation in decision-making [15, 16]. LHL patients ask fewer questions and take less control, but nevertheless do wish to take part in decision-making as much as other patients (e.g. [17]). The resulting unmet information needs constitute barriers to shared decision-making, whereas interventions to increase information exchange, openness and respect for a patient’s choice can act as facilitators [18]. Recognizing patients with LHL and adapting the organization of care, information provision, communication and decision-making to the wishes and needs of the patient are prerequisites to achieve desired person-centred palliative care and shared decision-making.

Many health care professionals, however, insufficiently check whether or not patients understand the information they provide, do not explore what the patient already knows and what information is still needed, and rarely discuss preferences for palliative or end-of-life care [14, 19]. In addition, many patients are unaware of their prognosis or the palliative nature of treatments [20–23]. Supporting tools (e.g. patient decision aids and question prompt sheets) and communication strategies (e.g. using short sentences, familiar words and allowing patients to record the conversation) are needed to improve this situation [19, 24].

Previous studies have revealed a relationship between health literacy and informed decision-making in several patient groups [16, 25–30]. The setting of palliative hospital-based care so far remains understudied [31, 32]. Because of the difficulties patients with LHL experience in communicating, understanding information and decision-making combined with the high demands posed on professionals regarding appropriate and effective communication and decision-making in palliative care, more insight is needed in available strategies and tools to support healthcare providers in their communication with patients with LHL in hospital-based palliative care.

The aim of this study is to summarize available strategies and tools for healthcare providers towards successful communication, information provision and/or shared decision-making in supporting patients with LHL in hospital-based palliative care in Western countries.

## Methods

### Aim

The aim of this study is to summarize available strategies and tools for healthcare providers (HCPs) towards successful communication, information provision and/or shared decision-making in supporting patients with LHL in hospital-based palliative care in Western countries.

### Design: scoping review

A scoping review was conducted. A scoping review is a literature review that is used when: 1) a narrow review question cannot be defined; 2) studies have employed a range of data collection and analysis techniques; 3) no prior synthesis has been undertaken on the topic; and 4) the reviewers are not going to assess the quality of the studies reviewed. With a scoping review the breadth of knowledge that is available about a particular topic is examined, therefore no quality constraints are applied [33].

### Search strategy

Databases PubMed, Embase, CINAHL, and PsycINFO were searched on August 14th 2017, by one author (MK). Keywords for the search were determined by two authors (JN and MK) after an initial broad search of the literature and based on the search strategy in a previous report about limited health literacy [34]. The search strategy in PubMed was adapted to the other databases. See Additional file 1 for the final search strategies.

Next, grey literature (e.g. thesis, reports, chapters, policy documents) were searched for in several online databases in November and December 2017 by one author (JN): Google Scholar, OpenGrey and CareSearch, using free text terms (e.g. palliative care, limited health literacy, communication, information-provision, shared decision-making, health care organization, professionals). See Additional file 2. We also contacted national experts in this field for possible additional literature. Finally, the references of included publications were checked for possible additional publications by one author (JN).

### Selection process

All database publications were entered in EndNote software and duplicates were removed. The database publications were independently reviewed by two authors (JN and MK). Firstly the publications were screened based on title and abstract. Secondly the remaining publications were reviewed full text. Disagreements were resolved by discussion between the authors. Grey literature, including literature received from national experts, was screened by one author (JN) based on title and abstract. The remaining full text articles were independently screened by two authors (JN and LvV). Disagreements were resolved by discussion between the authors.

Although we focus on hospital-based palliative care (i.e. both in- and outpatient hospital-based palliative care), we also included relevant studies from primary palliative care, as these studies may provide strategies and tools that are also useful in hospital-based care. In addition, to not narrow down our search we did not include the keywords ‘tools’ and ‘strategies’ beforehand. Post-hoc this was added to the inclusion criteria. The development of such ‘post hoc’ criteria is central to the scoping review process as it is unlikely that researchers will be able to identify parameters for exclusion at the beginning [33].

### Inclusion and exclusion criteria

The following inclusion criteria were defined:

1. Publication in English or Dutch
2. Publication year 2000 or later. This time frame was chosen to include up-to-date and still clinical relevant strategies and tools.
3. Study took place in a Western country.
4. Concerned patients (≥18 years) with limited health literacy, and/or their relatives, and their healthcare providers (include: literacy studies).
5. Study focused on communication and/or information-provision and/or shared decision-making
6. Study was set in palliative care [35]
7. Study took place in secondary and/or primary care
8. Described (the use of) strategies or tools for communication and/or information provision and/or shared decision-making by healthcare providers

Exclusion criteria were:

1. Terminal phase of care, if separate from palliative phase
2. Patients with severe cognitive impairment or psychiatric disorder (included: mild cognitive impairment, excluded: dementia)
3. Protocol of a study
4. Other (e.g. letter, conference abstract only, full text publication not found)

### Data extraction

A spreadsheet was created to chart the information that contributed to answering the research question: ‘Which communication strategies and tools are available for HCPs in supporting patients with LHL in hospital-based palliative care in Western countries?’ Data extraction of the included literature was done by one authors (JN) and checked by a second author (LvV). Disagreements were resolved by discussion between the authors.

## Results

### Flowchart

**Fig. 1:**
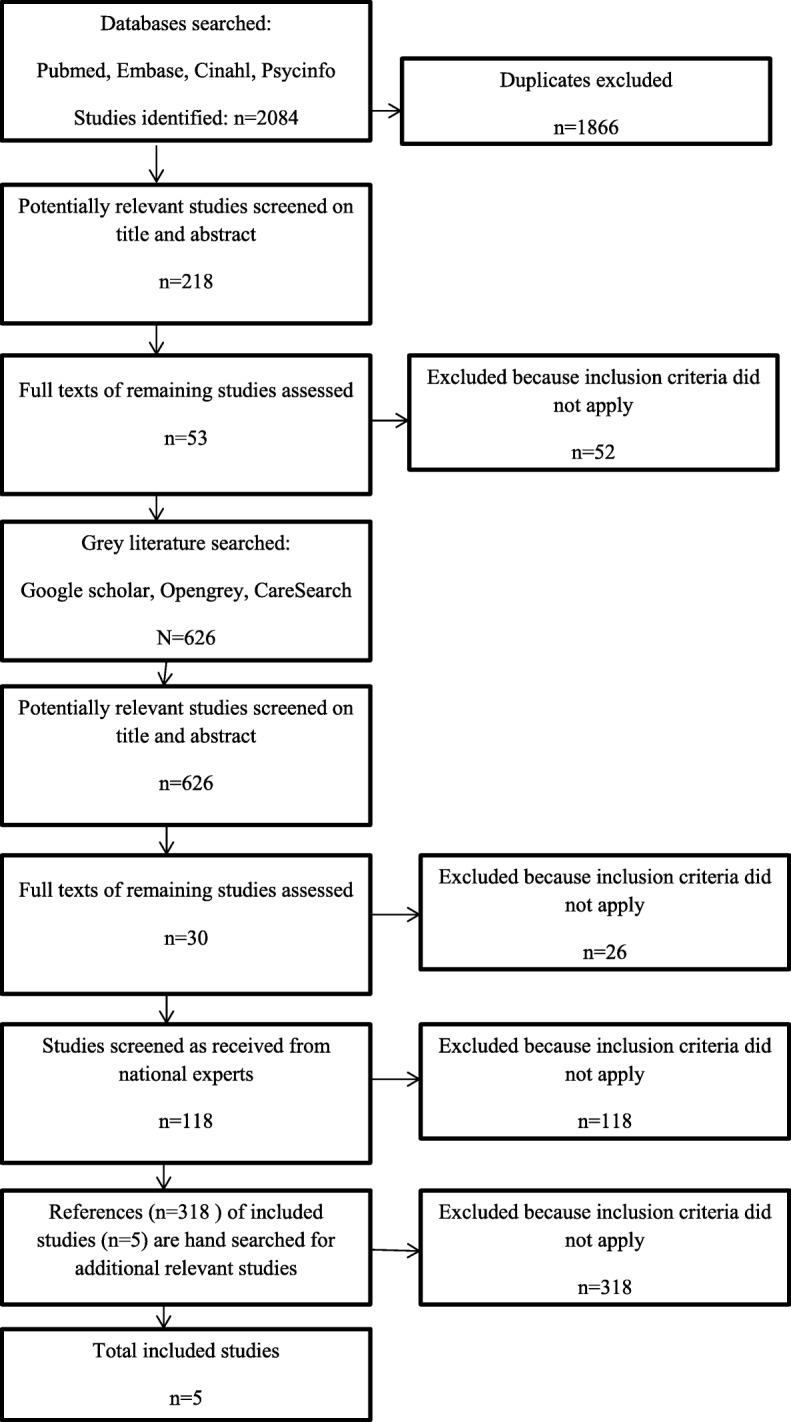
Flow chart of search strategy and results

As shown in Fig. 1, a total of 218 non-duplicate publications were identified from the databases. After selection, one publication remained for inclusion in this review.

In addition, grey literature revealed 626 publications, of which four publications were included.

National experts reported a total of 118 studies, no study was included.

Finally, we screened all references of the included publications, no publications were included.

### Study characteristics

In Table 1 an overview of the characteristics of the five included studies is given.

**Table 1:** Characteristics of the included studies

	First author, year, country	Healthcare provider	Patients	Setting/aim	Study design	Face-to-face - and/or written & online strategies/tools^a^	Other
1	Ache, 2009, USA [46]	Healthcare providers in general/not specified	Patients with low-literacy in palliative and end of life care	Internet websites of 5 nationally prominent US-based palliative care organisations were searched. A convenience sample of 15 patient education materials per palliative care organization was drawn (*n* = 75).	Review of patient information materials from the internet	Written & online strategies	–
2	Chou, 2015, USA [19]	Healthcare providers in general/not specified	Patients with limited health literacy in palliative care	Using a multilevel ecological framework to highlight connections between health literacy and palliative care across a wide variety of health areas, including: palliative care utilization; communication between providers, patients and other caregivers; self-care and caregiving outside the clinical setting	Overview of available strategies and tools to address health literacy in palliative care, derived from previous studies (Book chapter; no information about numbers of patients/ providers/ materials included)	Face-to-face, written & online strategies and tools	Also strategies and tools at patient, organizational, community and national policy level
3	Fage-Butler, 2015, USA [47]	Healthcare providers in general/ not specified	Patient and family centred care, with a focus on health literacy in palliative care	Three types of written & online palliative care communication were described: one-to one: provider responds to needs of individual patient (email); one-to-many: provider communicates to a group (end-of-life leaflets); many-to-many: a bank of providers share advice or responses to a group (online patient forums)	Overview of available written and online strategies to support the needs of palliative care patients, derived from previous studies (Book chapter; no information about numbers of patients/ providers/ materials included)	Written & online strategies	–
4	Kidd, 2014, New Zealand [45]	Healthcare providers in primary and specialist care. Also formal and informal community care providers	Maori patients and their extended family in palliative care, with a focus on health literacy	To understand health literacy in palliative care for Maori, their clinicians and community delivery of these services	Qualitative study based on interviews (*n* = 21 patients in palliative specialist care and their extended family; *n* = 6 providers/key informants); focus groups (*n* = 54 health professionals from different disciplines); written resource analysis (*n* = 42 written resources from participating palliative care providers); literature review (*n* = 27 articles included, most qualitative studies)	Face-to-face, written & online strategies	–
5	Rawlings, 2015, Australia [44]	Healthcare providers in general, focus on nurses	Patients with limited health literacy in palliative care	Australian website (CareSearch) for palliative care patients and providers, with evidence based, easy to read and understand information.	Clinical update & case study (review of CareSearch website) in palliative care (no information about numbers of patients/ providers/ materials included)	Face-to-face, written & online strategies	Also strategies at patient and organizational level

^a^Tools/strategies refer to interventions for healthcare providers for communicating with LHL patients in palliative care, including strategies and tools to support information provision and (shared) decision-making

Most studies were from the USA, had a qualitative nature and were aimed at healthcare providers in general, i.e. the type of provider was not specified. All studies took place in (secondary or primary) palliative care and were aimed at patients with limited (health) literacy.

### Strategies and tools

Most studies mentioned *strategies* for healthcare provider to support LHL patients in palliative care (see Tables 2 and 3). Table 2 provides an overview of the available *face-to-face strategies* and Table 3 describes the *written and online strategies* for healthcare providers in supporting LHL patients in hospital-based palliative care. The *face-to-face strategies* that were reported in several studies are: a) Teach-back method, a communication confirmation method used by HCPs to confirm whether a patient understands what is being explained, by asking the patient to tell what has been discussed. If a patient understands, they are able to “teach-back” the information accurately; b) Jargon free communication, i.e. the use of lay terminology; c) Slow down rate of speech, use short sentences and familiar words, limit provided information to a maximum of three main points when possible; d) Use patient navigators, i.e. trained culturally competent personnel who help patients and families address barriers to healthcare (see Table 2).

**Table 2:** Face-to-face strategies for healthcare providers (alphabetic order reference)

Strategy	References
Teach-back method	[19, 44]
Jargon free communication	[19, 44]
Slow down rate of speech, use short sentences and familiar words, limit provided information to a maximum of three main points when possible	[19, 44]
Use patient navigators	[19, 45]
Plan sufficient time for a consultation	[19]
Allow patients to record the consultation	[19]
Focus only on information most critical to patients’ decision-making, i.e. seek to understand the priorities and values of patients and their families and explain how they might best achieve their goals given the options available	[19]
Pay attention to communication about prognostic and treatment options, especially the numeric format of the information. Supplementing face-to-face communication about numeric data with graphs or other visual displays facilitates comprehension, especially the use of pictographs.	[19]
Incorporate health literacy in medical training	[19]
Group-based education programs on caregiving and coping with loss	[19]
Encourage patients to have a preferred support person present for important discussions	[19]
Employ specialized counsellors to improve communication with patients	[19]
Information should focus on actionable information relevant to patients concerns	[19]
Adopt universal precautions that reduce the cognitive burden placed on all patients and ensure the comprehension of key information, instead of viewing limited health literacy as the exception to the rule	[19]
Ability for patients to communicate 24/7 with a health professional, to ask questions and have their fears allayed	[45]
Establish respectful rapport with patients	[45]
Help with predicting future care needs of patients	[45]
Have regular meetings with people providing care to patients to discuss progress (both formal and informal care givers)	[45]
Being open and honest and advising patients of the reality of the situation	[45]
Early assessment of post-discharge needs	[44]
Tailor information to patients	[44]
Provide information about medication	[44]

**Table 3:** Written and online strategies for healthcare providers (alphabetic order reference)

Strategy	References
Provide clear, brief, jargon-free information (lay terminology) in a conversation style (active voice), supported by graphs, illustrations or visuals	[19, 44, 46]
Use large font size and ample white space	[19, 44, 46]
Develop and test materials with the help of members of the target population (i.e. patients with limited health literacy)	[19, 44, 47]
Use short sentences and paragraphs	[19, 44]
Use audio and video recordings as presentation materials, especially with complex issues as prognosis and treatment preferences	[19]
Materials should be linguistically and culturally sensitive	[19]
Tailor communication to patients’ health literacy level (e.g. terminology)	[47]
Healthcare ﻿providers should visit relevant patient forums to gain insight into patients’ needs	[47]
Inform palliative care patients about condition-related forums that may support their information and relational need	[47]
Engage in email communication with palliative care patients who express a wish for this, while bearing in mind the potential pitfalls associated with this medium	[47]
Use a ‘communication book’ to record what is happening (filled in by all healthcare providerss, patient and their significant others)	[45]
With respect to e-health literacy: use a checklist for consideration in the web environment	[44]

Chou and colleagues [19] also mentioned the use of *tools* in their book chapter. They mention ‘question prompt lists’ (QPS), i.e. structured lists of questions for the patient to ask their HCPs, as facilitators to patient-provider communication and to empower patients to discuss prognosis and end-of-life issues. In addition, ‘patient decision aids’(PDA), which contain structured and personalized information about treatment options, have the potential to facilitate communication and reducing decision burden according to these authors [19].

The *written and online strategies* described in several studies are: a) Provide clear, brief, jargon-free information (lay terminology) in a conversation style (active voice), supported by graphs, illustrations or visuals; b) Use large font size and ample white space; c) Develop and test materials with the help of members of the target population (i.e. patients with LHL); d) Use short sentences and paragraphs (see Table 3).

In Additional file 3 some examples of the reported strategies described in the Tables 2 and 3 are provided.

## Discussion

This scoping review showed that there are several face-to-face, written as well as online strategies available for healthcare providers to support communication, information provision and, to a lesser extent, decision-making in palliative care for patients with LHL. The *face-to-face strategies* that were reported in several studies were: teach-back method; jargon free communication; slow down rate of speech, use short sentences and familiar words, limit provided information to a maximum of three main points when possible, and the use of patient navigators. The *written and online strategies* described in several studies were: provide clear, brief, jargon-free information (lay terminology) in a conversation style (active voice), supported by graphs, illustrations or visuals; use large font size and ample white space; develop and test materials with the help of members of the target population and use short sentences and paragraphs. Two supporting tools were found: question prompt lists (QPS) and patient decision aids (PDA). However, a specific QPS or PDA being developed for and used in hospital-based palliative care to support patients with LHL was not mentioned [19]. In addition, these authors also point to a review that concludes that most PDAs are not designed and tested with patients with LHL and do not conform to literacy criteria [16].

Although we searched for strategies and tools for HCPs to support patients with LHL in hospital-based palliative care, the found strategies and tools appear not specific for the setting of palliative care. They seem helpful for HCPs to support the communication, information provision and decision making with patients with LHL in general. This does not mean that those strategies and tools are not useful in palliative care, but that more research is needed into the development and evaluation in this setting. As mentioned before, the high demands posed on professionals regarding appropriate and effective communication and decision-making in palliative care, combined with the difficulties patients with LHL experience in communicating, understanding information and decision-making suggests a challenge for research and practice.

In addition, from this scoping review we cannot determine if a particular strategy or tool is (more) effective for HCPs in supporting LHL patients in palliative care. It is not the aim of a scoping review to assess the quality of the included studies, but the breadth of knowledge that is available about this topic.

Adapting (effective) strategies and tools for HCPs in palliative care to the population of patients with LHL, or adapting (effective) interventions for patients with LHL to the setting of hospital-based palliative care, might be another approach to improve the communication making use of already available resources. For example, there are several communication tools developed in Belgium for HCPs in palliative care to support patients [36] and also several internationally initiatives take place for advanced care planning in palliative care (e.g. [14, 37]). However, these strategies and tools are not tested with or adapted to patients with LHL. In addition, there are several interventions who focus on patients with LHL [38, 39] or tools for professionals to support patients with LHL [40, 41]. Though, these interventions are not tested or designed for HCPs in hospital-based palliative care. Moreover, although a number of reading- and comprehension-assessment tools are available, there is debate whether or not these tools should be used clinically [42]. A study by Volandes et al. [43] found that video decision aids improved end-of-life decision making by decreasing uncertainty regarding subjects’ preferences in patients with dementia, especially for those with limited literacy. This also seems a promising tool for HCPs who support patients with LHL in hospital-based palliative care. However, it can still be difficult for HCPs to identify patients who have LHL and also recognize when these patients do not comprehend the information, as patients can hide their LHL from HCPs due to shame or feign understanding during conversations [19, 44].

### Strengths and limitations

In this scoping review we attempted to draw a picture of available strategies and tools for HCPs to support the communication with LHL patients in palliative care, without selecting studies on quality. A strong point is the broad scope of literature included, from several sources, and the careful review process with two reviewers. Another strength is the fact that, as far as we know, we are the first to conduct a scoping review on this topic. Some limitations should also be mentioned. Unfortunately, only five studies were available that fulfilled our criteria. We do not think that our search strategy was too narrow, but that only few studies are available on this topic. In addition, most studies had a qualitative nature. This stresses the need for future practical developments and research in this area. Furthermore, although we used an international definition of limited health literacy [3], we disregard the group of people who are temporarily limited health literate because of for example the shock and emotions of their diagnoses or a family loss. This group might be even larger than the group of people with LHL and could also benefit from the available strategies and tools we found. Finally, our focus was on hospital-based palliative care although we also included primary care based palliative care initiatives for healthcare providers. It is possible that the strategies found for primary care providers can not be (directly) used by providers in hospital-based care. However, most studies were aimed at HCPs in general and only one study specifically mentioned primary care providers [45]. Moreover, we consider adapting the communication strategy or tool to the needs of individual patients by HCPs as more important than the setting in which the care is provided.

Finally, we excluded studies about the terminal phase of care, if separate from the palliative phase. In theory, terminal care is different from palliative care but in practice these phases can overlap. Therefore, we did include studies that combined palliative and terminal care, as well as end-of-life care studies.

### Recommendations for practice and research

This study showed several available strategies, and to a lesser extent tools, for HCPs. As mentioned before, research into the development and evaluation of (the most) effective strategies and tools for HCPs to support patients with LHL in hospital-based palliative care research is needed. In addition, the effective strategies and tools should be implemented in practice by means of training and education. Learning and embedding new communication strategies is difficult and requires (extensive) training for HCPs.

The included studies also mentioned several recommendations or priorities for (future) practice and research. For example, Ache and colleagues [46] pointed to the necessity to revise or develop appropriate written low-literate patient education materials. Rawlings and Tieman [44] point out that to recognize and acknowledge that ‘there are large numbers of patients who do not read well or struggle to understand health information’ is vital. Chou and colleague [19] also stressed the importance of increasing access to and utilization of palliative care, for example by increasing the use of patient navigators or using patient narratives or ‘role-model stories’. In addition, emerging technologies and media (e.g. automated reminders, computerized agents, social media platforms) with user-centred design for future research and practice should be considered [19]. This also entails that researchers need to find out the specific features of technology-mediated innovations that patients and their families find useful and engaging [19]. Fage-Butler and Jensen [47] also mention the importance to ask patients and their caregivers for their perspectives, to meet their needs concerning written communication materials [47]. Finally, Chou et al. [19] recommend that researchers move beyond the cancer care setting, since most palliative research to date focus on cancer patients, including those with LHL. Indicating that providers and systems need to focus on individual patients and families rather than on a disease.

## Conclusions

To guarantee high quality person-centred palliative care, the role of health literacy should be considered. Recognizing LHL patients and adapting the organization of care, communication, information provision and decision-making to the wishes and needs of the patient are prerequisites to achieve desired person-centred palliative care and shared decision-making. Although there are several strategies available for HCPs to facilitate such communication, only few tools are offered for HCPs. Moreover, the strategies and tools appear not specific for the setting of palliative care, but seem helpful for HCPs to support the communication, information provision and decision making with patients with LHL in general. Future research should focus on which strategies or tools are (most) effective in supporting LHL patients in palliative care, and the implementation of these strategies and tools in practice.

## Additional files

Additional file 1: Search strategies for databases PubMed, Embase, CINAHL, and PsycINFO. This file contains the search strategies for databases PubMed, Embase, CINAHL, and PsycINFO. (DOCX 20 kb)

Additional file 2: Search strategies grey literature. This file contains the search strategies in grey literature: Google Scholar, OpenGrey and CareSearch. (DOCX 15 kb)

Additional file 3: Examples of reported strategies and tools. This file contains some examples of the reported strategies and tools in Tables 2 and 3. (DOCX 19 kb)

## Abbreviations

HCPs: Healthcare providers
LHL: Limited Health Literacy
PDA: Patient decision aid
QPS: Question prompt sheet

## References

[1] Sørensen K, Peilkan JM, Röthlin F, Ganahl K, Slonska Z, Doyle G, Fullam J, Kondilis B, Agrafiotis D, Uiters E, Falcon M, Mensing M, Tchamov K, van den Broucke S, Brand H, on behalf of the HLS-EU consortium (2015). Health literacy in Europe: comparative results of the European Health Literacy survey (HLS-EU). Eur J Pub Health.

[2] Heijmans M, Brabers A, Rademakers J (2018). Health Literacy in Nederland.

[3] Nielsen-Bohlman L, Panzer A, Kindig D. Health literacy: A prescription to end confusion. Institute of Medicine / San Francisco: the Academic Press; 2004. 25009856

[4] Sørenson K, Van den Broucke S, Fullam J, Doyle G, Pelikan J, Slonska Z (2012). for HLS-EU. Consortium Health Literacy Project Europe. Health Literacy and public health: a systematic review and integration of definitions and models. BMC Public Health.

[5] Murtagh FE, Bausewein C, Verne J, Groeneveld E, Kaloki YE, Higginson IJ (2014). How many people need palliative care? A study developing and comparing methods for population-based estimates. Palliat Med.

[6] Stuurgroep Passende Zorg in de laatste levensfase. Niet alles wat kan hoeft. Utrecht, 2015.

[7] Institute of Medicine. Dying in America Improving Quality and Honoring Individual Preferences Near the End of Life. Report brief. 2014. http://www.nationalacademies.org/hmd/~/media/Files/Report%20Files/2014/EOL/Report%20Brief.pdf. Accessed 25 July 2018.

[8] Gary TL, Batts-Turner M, Yeh HC, Hill-Briggs F, Bone LR, Wang NY (2009). The effects of a nurse case manager and a community health worker team on diabetic control, emergency department visits, and hospitalizations among urban African Americans with type 2 diabetes mellitus: a randomized controlled trial. Arch Intern Med.

[9] Clancy C (2009). Reengineering hospital discharge: a protocol to improve patient safety, reduce costs, and boost patient satisfaction. Am J Med Qual.

[10] Collins A, McLachlan S-A, Philip J (2018). Communication about palliative care: a phenomenological study exploring patient views and responses to its discussion. Pal Med..

[11] Henselmans I, van Laarhoven HWM, van der Vloodt J, de Haes HCJM, Smets EMA (2017). Shared decision making about palliative chemotherapy: a qualitative observation of talk about patients’ preferences. Pal Med..

[12] Perkins P, Barclay S, Booth S (2007). What are patients’ priorities for palliative care research? Focus group study. Pal Med..

[13] Perkins P, Booth S, Vowler SL, Barclay S (2008). What are patients’ priorities for palliative care research? – a questionnaire study. Pal Med..

[14] Jabbarian LJ, Zwakman M, van der Heide A, Kars MC, Janssen DJA, van Delden JJ (2018). Advance care planning for patients with chronic respiratory diseases: a systematic review of preferences and practices. Thorax..

[15] Kim SP, Knight SJ, Tomori C, Colella KM, Schoor RA, Shih L (2001). Health literacy and shared decision making for prostate cancer patients with low socioeconomic status. Cancer Investig.

[16] McCaffery KJ, Holmes-Rovner M, Smith SK, Rovner D, Nutbeam D, Clayman ML (2013). Addressing health literacy in patient decision aids. BMC Med Inform Decis Mak.

[17] Katz MG, Jacobson TA, Veledar E, Kripalani S (2007). Patient literacy and question-asking behaviour during the medical encounter: a mixed-methods analysis. J Gen Intern Med.

[18] Bélanger E, Rodriguez C, Groleau D (2011). Shared decision-making in palliative care: a systematic mixed studies review using narrative synthesis. Pal Med..

[19] Chou W-Y S, Gaysynsky A, Persoskie A, Wittenberg E, Ferrell BR, Goldsmith J, Smith T, Ragan SL, Glajchen M, Handzo G (2015). Chapter 12: Health literacy and communication in palliative care. Textbook of palliative care communication.

[20] Fried TR, Bradley EH, O’Leary MA (2003). Prognosis communication in serious illness: perceptions of older patients, caregivers, and clinicians. J Am Geriatr Soc.

[21] Hancock K, Clayton JM, Parker SM, Walder S, Butow PN, Carrick S (2007). Discrepant perceptions about end-of-life communication: a systematic review. J Pian Symptom Manage.

[22] Slort W, Schweitzer BPM, Blankenstein AH, Abarshi EA, Riphagen II, Echteld MA, Aaronseon NK, van der Horst HE, Deliens L (2011). Perceived barriers and facilitators for general practitioner- patient communication in palliative care: a systematic review. Pal Med.

[23] Yennurajalingam S, Rodrigues LF, Shamieh O, Tricou C, Filber M, Naing K (2018). Perception of curability among advanced cancer patients: an international collaborative study. Oncologist.

[24] Stacey D, Légaré F, Lewis K, Barry MJ, Bennett CL, Eden KB, et al. Decision aids for people facing health treatment or screening decisions. Cochrane Database of Systematic Reviews 2017; Issue 4. DOI: 10.1002/14651858.CD001431.pub5. 10.1002/14651858.CD001431.pub5 PMC6478132 28402085

[25] Seo J, Goodman MS, Politi M, Blanchard M, Kaphingst KA (2016). Effect of Health Literacy on decision-making preferences among medically underserved patients. Med Decis Mak.

[26] Barton JL, Trupin L, Tonner C, Imboden J, Katz P, Schillinger D (2014). English language proficiency, health literacy, and trust in physician are associated with shared decision making in rheumatoid arthritis. J Rheumatol.

[27] Goggins KM, Wallston KA, Nwosu S, Schildcrout JS, Castel L, Kripalani S (2014). Health literacy, numeracy, and other characteristics associated with hospitalized patients' preferences for involvement in decision making. J Health Commun.

[28] Smith SK, Simpson JM, Trevena LJ, McCaffery KJ (2014). Factors associated with informed decisions and participation in bowel cancer screening among adults with lower education and Literacy. Med Decis Mak.

[29] Yin HS, Dreyer BP, Vivar KL, MacFarland S, van Schaick L, Mendelsohn AL (2012). Perceived barriers to care and attitudes towards shared decision-making among low socioeconomic status parents: role of health literacy. Acad Pediatr.

[30] Naik AD, Street RL, Castillo D, Abraham NS (2011). Health literacy and decision making styles for complex antithrombotic therapy among older multimorbid adults. Patient Educ Couns.

[31] Baars JE, van Dulmen AM, Velthuizen ME, van Riel E, Ausems MGEM (2017). Breast cancer genetic counseling among Dutch patients from Turkish and Moroccan descent: participation determinants and perspectives of patients and healthcare professionals. J Community Genet..

[32] Van der Giessen JAM, van Riel E, Veldhuizen ME, van Dulmen AM, Ausems MGEM (2017). Referral to cancer genetic counseling: do migrant status and patients’ educational background matter?. J Community Genet.

[33] Crooks V, Kingsbury P, Snyder J, Johnston R (2010). What is known about the patient's experience of medical tourism? A scoping review. BMC Health Services Research.

[34] Heijmans M, Zwikker H, van der Heide I, Rademakers J (2016). NIVEL Kennisvraag 2016: Zorg op maat. Hoe kunnen we de zorg beter later aansluiten bij mensen met lage gezondheidsvaardigheden?.

[35] WHO. WHO definition of palliative care, 2002. http://www.who.int/cancer/palliative/definition/en/ Accessed 10 Aug 2017.

[36] Instrumenten voor zorgprofessionals (Tools for healthcare professionals). End-of-Life Care Research Group. http://www.endoflifecare.be/instrumenten-voor-zorgprofessionals. Accessed 15 Aug 2018.

[37] Voss H, Vogel A, Wagemans AMA, Francke AL, Metsemakers JFM, Courtens AM (2017). Advance care planning in palliative care for people with intellectual disabilities: a systematic review. J Pain Symptom Manag.

[38] Basu A, Brinson D, Ali W, Smartt P (2010). Interventions to mitigate the effects of low health literacy: a systematic review of the literature. HSAC Report.

[39] Sudore RL, Schillinger D (2009). Interventions to improve care for patients with limited health literacy. JCOM..

[40] Weis BD. Health literacy and patient safety: help patients understand. Manual for clinicians. Second edition. American Medical Association Foundation and American Medical Association, 2007.

[41] AHRQ Health Literacy Universal Precautions Toolkit. 2nd edition. Agency for Healthcare Research and Quality https://www.ahrq.gov/professionals/quality-patient-safety/quality-resources/tools/literacy-toolkit/index.html. Accessed 1 Aug 2018.

[42] Cornett S (2009). Assessing and addressing Health Literacy. OJIN: The Online Journal of Issues in Nursing.

[43] Volandes AE, Paasche-Orlow M, Gillick M, Cook EF, Shaykevich S, Abbo ED (2008). Health literacy not race predicts end-of-life care preferences. J Pal Med.

[44] Rawlings D, Tieman J (2015). Patient and carer information: can they read and understand it? An example from palliative care. Clinical update. Aust Nurs Midwifery J.

[45] Kidd JD, Reid S, Collins N, Gibbons V, Black S, Blundell R, Peni T, Ahu H. Kia Mau te Kahu Whakamauru: health literacy in palliative care. The University of Auckland. https://cdn.auckland.ac.nz/assets/fmhs/faculty/ABOUT/newsandevents/docs/Health%20literacy%20in%20palliative%20care%20report%20final.pdf. Accessed 20 Dec 2017.

[46] Ache KA, Wallace LS (2009). Are end-of-life patient education materials readable?. Pal Med.

[47] Fage-Butler AM, Jensen MN, Wittenberg E, Ferrell BR, Goldsmith J, Smith T, Ragan SL, Glajchen M, Handzo G (2015). Chapter 13: Patient- and family-centered written communication in the palliative care setting. Textbook of palliative care communication.

