# Supplementary material for: Towards appropriate information provision for and decision-making with patients with limited health literacy in hospital-based palliative care in Western countries: a scoping review into available communication strategies and tools for healthcare providers
Source: BMC Palliat Care. 2019 Apr 12;18:37. doi: 10.1186/s12904-019-0421-x (PMC6461806; doi:10.1186/s12904-019-0421-x)
Supplement: Supplementary file 1 — Search strategies for databases PubMed, Embase, CINAHL, and PsycINFO. This file contains the search strategies for databases PubMed, Embase, CINAHL, and PsycINFO. (DOCX 20 kb) [file 12904_2019_421_MOESM1_ESM.docx]

**Additional file 1. Search strategies for databases PubMed, Embase, CINAHL, and PsycINFO**

*Search strategy: Pubmed, 14 August 2017*

**Blok A: health literacy**

("Health Literacy"[Mesh] OR "Health Literacy"[tiab] OR “Information Literacy”[Mesh] OR "health competence"[tiab] OR “Health Knowledge, Attitudes, Practice”[Mesh] OR Literacy[Mesh] OR literacy[tiab] OR literate[Tiab] OR illiteracy[tiab] OR “reading proficiency”[tiab] OR “reading skills”[tiab] OR “writing proficiency”[tiab] OR “writing skills”[tiab]) AND (Adult[Mesh] OR Adult*[tiab] OR patients[Mesh] OR patient*[tiab])

**Blok B1: health care providers, institutions**

Health Personnel[Mesh] OR *care prov*[tiab] OR caregiver*[tiab] OR care giver*[tiab] OR General Practitioners[Mesh] OR general pract*[tiab] OR Physicians[Mesh] OR physician*[tiab] OR doctor*[tiab] OR Nurses[Mesh] OR nurs*[tiab] OR profession*[tiab] OR Physician assistants[Mesh] OR Hospitals[Mesh] OR hospital*[tiab] OR General Practice[Mesh] OR organization*[tiab] OR organisation* [tiab] OR Health Services[Mesh] OR health service*[tiab]

**Blok B2: palliative care**

(“Palliative Care”[Mesh] OR “Palliative care” [tiab] OR “palliative*”[tiab] OR “Hospice and palliative care nursing”[Mesh] OR “Terminal Care”[Mesh] OR “Terminal Care”[tiab] OR “end of life”[tiab])

**Blok C: Competences and skills**

(Professional Competence[Mesh] OR Clinical Competence[Mesh] OR competen*[tiab] OR Social Skills[Mesh] OR skill*[tiab] OR able[tiab] OR abilit*[tiab] OR Practice Guidelines as Topic[Mesh] OR Standard of Care[Mesh] OR guideline*[tiab] OR standard*[tiab])

**Blok D: Communication and decision making**

(Health Communication[mesh] OR “health communication"[tiab] OR communication[Mesh] OR communicat*[tiab] OR Communication Barriers[Mesh] OR Decision Making[Mesh] OR “shared decision making”[tiab] OR dialog*[tiab] OR convers*[tiab] OR talk*[tiab] OR interlocution*[tiab] OR deliberat*[tiab] OR counsel*[tiab] OR conjunct*[tiab] OR conjoint[tiab] OR “partners in care”[tiab] OR “partner in care”[tiab] OR interact*[tiab] OR “Clinical Decision Making”[Mesh] OR “patient preference”[Mesh])

**Blok E: Information dissemination**

(Information Dissemination[Mesh] OR consumer health information[Mesh] OR information[tiab] OR information material*[tiab] OR "patient education as topic"[mesh] OR "patient education"[tiab] OR e-health[tiab] OR teach-back communication[Mesh] OR teach-back*[tiab])

**Blok F NOT**

Resident*[tiab] OR adolescent*[tiab] OR child*[tiab] OR student*[tiab]

Search combined:

(A & B & C) & (D / E) (B1 & B2) = B ((A & B & C) & (D / E)) NOT F

*Search strategy: Embase, 14 August 2017*

**Blok A: health literacy**

“health literacy”/de OR “health literacy”:ab,ti OR “information literacy”/de OR “health competence”:ab,ti OR “health knowledge”:ab,ti OR literacy/de OR literacy:ab,ti OR literate:ab,ti OR illiteracy:ab,ti OR “reading proficiency”:ab,ti OR “reading skills”:ab,ti OR “writing proficiency”:ab,ti OR “writing skills”:ab,ti AND (adult/de OR adult*:ab,ti OR patients/de OR patient*:ab,ti)

**Blok B1: health care providers, institution**

“health care personnel”/de OR “care prov*”:ab,ti OR caregiver*:ab,ti OR “care giver*”:ab,ti OR “general practitioner”/de OR “general pract*”:ab,ti OR physician/de OR physician*:ab,ti OR doctor*:ab,ti OR nurse/de OR nurs*:ab,ti OR profession*:ab,ti OR “physician assistant”/de OR hospital/de ORhospital*:ab,ti OR “general practice”/de OR organization*:ab,ti OR organization*:ab,ti OR “health service”/de OR “health service”:ab,ti

**Blok B2: palliative care**

“palliative nursing”/de OR “palliative care”:ab,ti OR “palliative*”:ab,ti OR “terminal care”/de OR “terminal care”:ab,ti OR “end of life”:ab,ti

**Blok C: Competences and skills**

“professional competence”/de OR “clinical competence”/de OR competen*:ab,ti OR “social competence”/de OR skill*:ab,ti OR able:ab,ti OR abilit*:ab,ti OR “practice guideline”/de OR “health care quality”/de OR guideline*:ab,ti OR standard*:ab,ti

**Blok D: Communication and decision making**

“health communication”:ab,ti OR “interpersonal communication”/de OR communicat*:ab,ti OR “communication barrier”/de OR “decision making”/de OR “shared decision making”:ab,ti OR dialog*:ab,ti OR convers*:ab,ti OR talk*:ab,ti OR interlocution*:ab,ti OR deliberat*:ab,ti OR counsel*:ab,ti OR conjunct*:ab,ti OR conjoint:ab,ti OR “partners in care”:ab,ti OR “partner in care”:ab,ti OR interact*:ab,ti OR “clinical decision making”/de OR “patient preference”/de

**Blok E: Information dissemination**

“information dissemination”/de OR “consumer health information”/de OR information:ab,ti OR “information material*”:ab,ti OR “patient education”/de OR “patient education”:ab,ti OR e-health:ab,ti OR “teach-back communication”:ab,ti OR “teach-back*”:ab,ti

**Blok F: NOT**

Resident*:ab,ti OR adolescent*:ab,ti OR child*:ab,ti OR student*:ab,ti

*Search strategy: CINAHL, 14 August 2017*

**Blok A: health literacy**

MH “Health literacy” OR TI “health literacy” OR AB “health literacy” OR MH “information literacy” OR TI “health competence” OR AB “health competence” OR MH health OR MH health knowledge, attitudes, practice OR MH literacy OR TI literacy OR AB literacy OR TI literate OR AB literate OR TI illiteracy OR AB illiteracy OR TI “reading proficiency” OR AB “reading proficiency” OR TI “reading skills” OR AB “reading skills” OR TI “writing proficiency” OR AB “writing proficiency” OR TI “writing skills” OR AB “writing skills” AND (MH adult OR TI adult* OR AB adult* OR MH patients OR TI patient* OR AB patient*)

**Blok B1: health care providers, institutions**

MH “health personnel” OR TI “care prov*” OR AB “care prov*” OR TI “care giver” OR AB “care giver” OR MH “general practitioners” OR TI “general pract*” OR AB “general pract*” OR MH physicians OR TI physician OR AB physician OR TI doctor* OR AB doctor* OR MH nurses OR TI nurs* OR AB nurs* OR TI profession* OR AB profession* OR MH “physician assistant” OR MH hospitals OR TI hospital* OR AB hospital* OR MH “general practice” OR TI organization* OR AB organization* OR TI organisation* OR AB organisation* OR MH “health services” OR TI “health service*” OR AB “health service*”

**Blok B2: palliative care**

MH “palliative care” OR TI “palliative care” OR AB “palliative care” OR TI palliative* OR AB palliative* OR MH “hospice and palliative care nursing” OR MH “terminal care” OR TI “terminal care” OR AB “terminal care” OR TI “end of life” OR AB “end of life”

**Blok C: Competences and skills**

MH “professional competence” OR MH “clinical competence” OR TI competen* OR AB competen* OR MH “social skills” OR TI skill* OR AB skill* OR TI able OR AB able OR TI abilit* OR AB abilit* OR MH “practice guidelines as topic OR MH “standard of care” OR TI guideline* OR AB guideline* OR TI stanrd* OR AB standard*

**Blok D: Communication and decision making**

MH “health communication” OR TI “health communication” OR AB “health communication” OR MH communication OR TI communicat* OR AB communicat OR MH “communication barriers” OR MH “decision making” OR TI “shared decision making” OR AB “shared decision making” OR TI dialog* OR AB dialog* OR TI talk* OR AB talk* OR TI interlocution* OR AB interlocution* OR TI deliberat* OR AB deliberat* OR TI counsel* OR AB counsel* OR TI conjunct OR AB conjunct* OR TI conjoint OR AB conjoint OR TI “partners in care” OR AB “partners in care” OR TI “partner in care” OR AB “partner in care” OR TI interact* OR AB interact* OR MH “clinical decision making” OR MH “patient preference”

**Blok E: Information dissemination**

MH “information dissemination” OR MH “consumer health information” OR TI information OR AB information OR TI “information material” OR AB “information material” OR MH “patient education as topic” OR TI “patient education” OR AB “patient education” OR TI e-health OR AB e-health OR MH “teach-back communication” OR TI “teach-back*” OR AB “teach-back*”

**Blok F: NOT**

TI resident* OR AB resident* OR TI adolescent* OR AB adolescent* OR TI child* OR AB child* OR TI student* OR AB student*

*Search strategy: PsycINFO, 14 August 2017*

**Blok A: health literacy**

Health literacy.mh. OR health literacy.ti,ab. OR information literacy.mh. Or health competence.ti,ab. OR health.mh. OR health knowledge, attitudes, practice.mh. OR literacy.mh. OR literacy.ti,ab. OR literate.ti,ab. OR illiteracy.ti,ab. OR reading proficiency.ti,ab. OR reading skills.ti,ab. OR writing proficiency.ti,ab. OR writing skills.ti,ab. AND Adult.mh. OR Adult*.ti,ab. OR patients.mh. OR patient*.ti,ab.

**Blok B1: health care providers, institution**

Health personnel.mh. OR care prov*.ti,ab. OR caregiver*.ti,ab. OR care giver*.ti,ab. OR general practitioners.mh. OR general pract*.ti,ab. OR physicians.mh. OR physician*.ti,ab. OR doctor.ti,ab. OR nurses.mh. OR nurs*.ti,ab. OR profession*.ti,ab. OR physician assistant.mh. OR hospitals.mh. OR hospital*.ti,ab. OR general practice.mh. OR organization*.ti,ab. OR organisation*.ti,ab. OR health services.mh. OR health service*.ti,ab.

**Blok B2: palliative care**

palliative care.mh. OR palliative care.ti,ab. OR palliative*.ti,ab. OR terminal care.mh. OR terminal care.ti,ab. OR end of life.ti,ab.

**Blok C: Competences and skills**

Professional competence.mh. OR clinical competence.mh. OR competen*.ti,ab. OR social skills.mh. OR skill*.ti,ab. OR able.ti,ab. OR abilit*.ti,ab. OR practice guidelines as topic.mh. OR standard of care.mh. OR guideline*.ti,ab. OR standard.ti,ab.

**Blok D: Communication and decision making**

Health communication.mh. OR health communication.ti,ab. OR communication.mh. OR communicat.ti,ab. OR communication barriers.mh. OR decision making.mh OR shared decision making.ti,ab. Or dialog*.ti,ab. OR convers*.ti,ab. OR talk*.ti,ab. OR interlocution*.ti,ab. OR deliberat*.ti,ab. OR counsel*.ti,ab. OR conjunct*.ti,ab. OR conjoint.ti,ab. OR partners in care.ti,ab. OR partner in care.ti,ab. OR partner*.ti,ab. OR team*.ti,ab. OR interact.ti,ab. OR clinical decision making.mh OR patient preference.mh

**Blok E: Information dissemination**

Information dissemination.mh. OR consumer health information.mh. OR information.ti,ab. OR information material*.ti,ab. OR patient education as topic.mh. OR patient education.ti,ab. OR e-health.ti,ab. OR teach-back communication.mh. OR teach-back*.ti,ab.

**Blok F: NOT**

Resident*.ti,ab. OR adolescent*.ti,ab. OR child*.ti,ab. OR student*.ti,ab.
