# Supplementary material for: Towards appropriate information provision for and decision-making with patients with limited health literacy in hospital-based palliative care in Western countries: a scoping review into available communication strategies and tools for healthcare providers
Source: BMC Palliat Care. 2019 Apr 12;18:37. doi: 10.1186/s12904-019-0421-x (PMC6461806; doi:10.1186/s12904-019-0421-x)
Supplement: Supplementary file 2 — Search strategies grey literature. This file contains the search strategies in grey literature: Google Scholar, OpenGrey and CareSearch. (DOCX 15 kb) [file 12904_2019_421_MOESM2_ESM.docx]

**Additional file 2. Search strategy grey literature**

*Google scholar, 30 November 2017*

Free text: palliative care limited health literacy communication information-provision shared decision making health care organization professionals

The following boxes were ticked:

Custom range: 2000-2017

Search only in the languages: Dutch and English

No patents and quotes.

*CareSearch, 6 December 2017*

Search grey literature

<https://www.caresearch.com.au/caresearch/tabid/523/Default.aspx>

Search Terms: ((((((palliative care) AND limited health literacy) AND communication) AND information-provision) AND shared decision making) AND care organization) AND professionals

Type of literatures: All types (conference abstract, journal articles, thesis, research studies, other grey, quality programs, national programs)

Publication year: from 2000 to 2017

Items found: 0

Search strategy changed:

Search terms: (palliative care) AND limited health literacy

Type of literatures: All types (conference abstract, journal articles, thesis, research studies, other grey, quality programs, national programs)

Publication year: from 2000 to 2017

*OpenGrey, 6 December 2017*

<http://www.opengrey.eu/>

Search: palliative care limited health literacy communication information-provision shared decision making health care organization professionals

Language: English

Results: 0

Search strategy changed: palliative care AND limited health literacy AND communication AND information-provision AND shared decision making AND health care organization AND professionals.

Results: 0

Search strategy changed: Palliative care AND limited health literacy

Results: 0

Search strategy changed: Palliative care limited health literacy

Results: 0

Search strategy changed: Palliative care health literacy

Results: 0

Search strategy changed: Palliative care literacy

Results: 0

Final search strategy :

Search: Palliative care

Language: English

(included studies were also before the year 2000 as we could not select on publication year)
