# Supplementary material for: Towards appropriate information provision for and decision-making with patients with limited health literacy in hospital-based palliative care in Western countries: a scoping review into available communication strategies and tools for healthcare providers
Source: BMC Palliat Care. 2019 Apr 12;18:37. doi: 10.1186/s12904-019-0421-x (PMC6461806; doi:10.1186/s12904-019-0421-x)
Supplement: Supplementary file 3 — Examples of reported strategies and tools. This file contains some examples of the reported strategies and tools in Tables 2 and 3. (DOCX 19 kb) [file 12904_2019_421_MOESM3_ESM.docx]

**Additional file 3. Examples of reported strategies and tools**

- Chou et al. [19] mention two studies in which video formats may enhance understanding of end-of-life care options, including palliative care. For example, to elicit patient preference for care one study presented a verbal description of the disease and three available care options to participants, and also showed a video depicting a patient with that disease and the different treatments associated with the three available care options to the same participants. After watching the video more participants (89% versus 50%) preferred comfort care, also in the group of minorities and lower educated people.
- Chou et al.[19] mention examples of previous studies that found to facilitate patient understanding by providing: probabilistic information presented as frequency (e.g. ‘4 out of 10 patients’’) instead of presented as percentage (e.g. ‘40% of patients’); when comparing treatment options, risks described in terms of their absolute levels (e.g. ‘the number of patients who experience the side effect would increase from 1 in 1,000 to 2 in 1,000) are preferable to those described in relative levels (‘the risk would double’); and using a consistent denominator when comparing risks (e.g. ‘1 in 100’ vs. ‘10 in 100’).
- Fage-Butler et al. [37] provide the example of end-of-life leaflets, which should be written in a way that promotes understanding and meet palliative care patients’ physical needs (indirectly), intellectual, emotional and spiritual needs, and access to information.
- Kidd and colleagues [38] formulated specific health literacy demands concerning three topics for HCPs: medicines, providing care to patients and navigating the palliative care system (knowledge & tasks). For example with respect to ‘providing care to patients’ some tasks are: work in partnership with their significant other(s) and other health professionals; monitor patient’s physical, mental and spiritual wellbeing.
- Rawlings et al. [39] mention a checklist, with 10 items, for consideration in the web environment, e.g. what readability levels have been set and how are they decided; are images appropriate to reinforce the message; could the message be delivered in another format e.g. video.
